# Fluoroquinolone stewardship at a community health system: A decade in review

**DOI:** 10.1017/ash.2022.326

**Published:** 2022-11-16

**Authors:** Elena A. Swingler, Matthew Song, Sarah E. Moore, Brian C. Bohn, Paul S. Schulz, Alan D. Junkins, Ashley M. Wilde

**Affiliations:** 1 Norton Infectious Diseases Institute, Norton Healthcare, Louisville, Kentucky; 2 Department of Pharmacy, Barnes-Jewish Hospital, St. Louis, Missouri; 3 Department of Microbiology, Norton Healthcare, Louisville, Kentucky

## Abstract

**Objective:**

To describe inpatient fluoroquinolone use and susceptibility data over a 10-year period after the implementation of an antimicrobial stewardship program (ASP) led by an infectious diseases pharmacist starting in 2011.

**Design:**

Retrospective surveillance study.

**Setting:**

Large community health system.

**Methods:**

Fluoroquinolone use was quantified by days of therapy (DOT) per 1,000 patient days (PD) and reported quarterly. Use data are reported for inpatients from 2016 to 2020. Levofloxacin susceptibility is reported for *Pseudomonas aeruginosa* and *Escherichia coli* for inpatients from 2011 to 2020 at a 4 adult-hospital health system.

**Results:**

Inpatient fluoroquinolone use decreased by 74% over a 5-year period, with an average decrease of 3.45 DOT per 1,000 PD per quarter (*P* < .001). Over a 10-year period, inpatient levofloxacin susceptibility increased by 57% for *P. aeruginosa* and by 15% for *E. coli*. *P. aeruginosa* susceptibility to levofloxacin increased by an average of 2.73% per year (*P* < .001) and had a strong negative correlation with fluoroquinolone use, r = −0.99 (*P* = .002). *E. coli* susceptibility to levofloxacin increased by an average of 1.33% per year (*P* < .001) and had a strong negative correlation with fluoroquinolone use, r = −0.95 (*P* = .015).

**Conclusions:**

A substantial decrease in fluoroquinolone use and increase in *P. aeruginosa* and *E. coli* levofloxacin susceptibility was observed after implementation of an antimicrobial stewardship program. These results demonstrate the value of stewardship services and highlight the effectiveness of an infectious diseases pharmacist led antimicrobial stewardship program.

Antimicrobial resistance is a global public health threat mainly driven by the misuse of antimicrobials.^1^ Fluoroquinolone antibiotics have historically been prescribed for a wide variety of infections, including mild, uncomplicated infections for which alternatives may be preferred.^2^ Consequently, increasing antimicrobial resistance has been reported, particularly among Enterobacterales and *Pseudomonas* spp.^3–5^ Antimicrobial resistance greatly limits empiric and definitive treatment options for potentially serious infections. For example, for *Pseudomonas* spp infections, fluoroquinolones are the only antibiotic class currently available for enteral administration. Fluoroquinolone exposure has not only been associated with fluoroquinolone-resistant strains but also with methicillin-resistant *Staphylococcus aureus* and extended-spectrum β-lactamase–producing organisms.^6–8^

In addition to driving resistance, fluoroquinolones have been associated with serious and potentially permanent musculoskeletal and central nervous system adverse events resulting in regulatory action by the Food and Drug Administration.^9^ Their use also carries a moderate to high risk of *Clostridioides difficile* infection compared with other antibiotic classes, and fluoroquinolones have been linked to the emergence and spread of the hypervirulent 027/BI/NAP1 strain.^10–12^

In response to the concerns outlined above, many health systems have employed antimicrobial stewardship interventions to reduce fluoroquinolone use, antimicrobial resistance, and *C. difficile* infections. Interventions usually consist of formulary restriction and prospective audit and feedback, sometimes in conjunction with education and guidelines for optimal antibiotic use.^13^ Evidence to support fluoroquinolone-targeted interventions is heterogeneous regarding intervention types, duration, and outcomes measured.^13^ Overall, stewardship interventions are supported by favorable outcomes of reductions in fluoroquinolone use, resistance, and *C. difficile* infection rates.^13^

In this report, we describe fluoroquinolone use and susceptibility data over a 10-year period at a large community health system with an antimicrobial stewardship program (ASP).

## Methods

This retrospective surveillance study was conducted at Norton Healthcare, a large, integrated health system of 4 adult hospitals licensed for ∼1,600 beds in Kentucky. Inpatient fluoroquinolone use and susceptibility data were analyzed in the context of a growing ASP.

### Antimicrobial stewardship program

The current ASP at our health system started in 2011 with 1 full-time infectious diseases pharmacist and 1 part-time infectious diseases physician. Initially, the ASP focused on formulary optimization, guidelines for optimal antimicrobial use, order-set updates, and provider and pharmacist education. Fluoroquinolone use was addressed primarily through order-set revisions to discourage routine use. After maximizing the antimicrobial stewardship benefit that could be achieved at the system level, the infectious diseases pharmacist in charge of the ASP focused on providing prospective audit and feedback for patients on broad-spectrum antimicrobials, including fluoroquinolones. In 2017, the ASP expanded to include 4 full-time infectious diseases pharmacists (1 at each hospital within the health system) and was able to consistently provide prospective audit and feedback for a larger number of patients.^14^ The ASP continues to be supported by a part-time infectious diseases physician leader, but prospective audit and feedback is performed by the pharmacists. Formulary restriction of fluoroquinolones was not utilized at our health system.

### Antibiotic use

Inpatient levofloxacin and ciprofloxacin antibiotic use is expressed as days of therapy (DOT) per 1,000 patient days (PD) and is reported quarterly. DOT was calculated using administration records obtained from the electronic health record. Inpatient fluoroquinolone use data are reported for 2016 to 2020. Data were not readily retrievable for inpatient use before 2016.

### Microbiology

Levofloxacin susceptibility data for *P. aeruginosa* and *E. coli* were obtained from the health system’s antibiograms, which were collated annually for each hospital. Antibiograms were created using only the first isolate of a given species per patient per year and included nonsurveillance samples from inpatient and emergency department patients. Antibiotic susceptibility testing was performed on the MicroScan WalkAway system (Beckman Coulter, Brea, CA). Susceptibility was reported using the Clinical and Laboratory Standards Institute (CLSI) breakpoints established prior to 2019. Adult hospital susceptibility rates were aggregated and weighted according to the number of isolates per hospital per year. The results were reported as the proportion of fluoroquinolone-susceptible isolates among all clinical isolates for each species. Microbiologic data were reported for the period of 2010 to 2020 for inpatients.

### Statistical analysis

Line graphs were produced to show changes in fluoroquinolone DOT per 1,000 PD and changes in susceptibility to levofloxacin in *E. coli* and *P. aeruginosa* over time. Linear regression was used to produce trend lines and to describe changes over time. Beta coefficients, 95% confidence intervals, and *P* values were reported.

Scatter plots were produced to plot fluoroquinolone DOT per 1,000 PD versus susceptibility to levofloxacin in *E. coli* and *P. aeruginosa*. Pearson correlation coefficients were produced to assess ecological associations between DOT per 1,000 PD and susceptibilities. The Pearson correlation coefficient, r, and *P* values were reported. All statistical analyses were performed in R version 4.1.2 software (R Foundation for Statistical Computing, Vienna, Austria). *P* values < .05 were deemed statistically significant.

## Results

### Antibiotic use

**Fig. 1.**
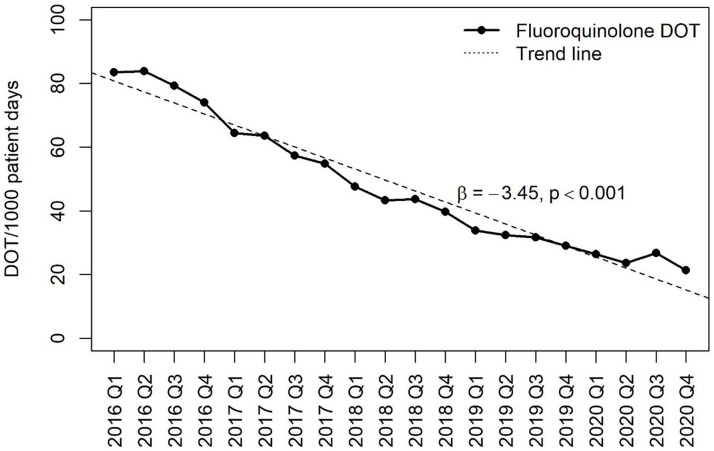
Fluoroquinolone use in adult inpatients from 2016 to 2020.

Inpatient fluoroquinolone use decreased from 83.5 DOT per 1,000 PD in quarter 1 of 2016 to 21.4 DOT per 1,000 PD in quarter 4 of 2020, representing a 74% decrease over a 5-year period. Fluoroquinolone use decreased over time by an average of 3.45 DOT per 1,000 PD per quarter, β = −3.45 (95% CI, −3.80 to −3.09; *P* < .001) (Fig. 1).

### Microbiology

**Fig. 2.**
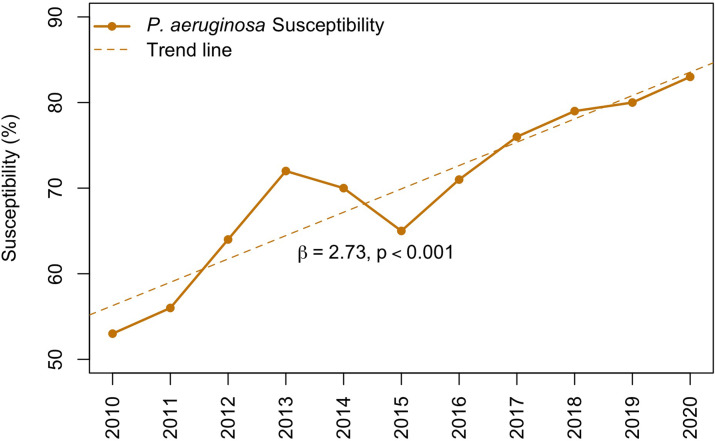
*P. aeruginosa* levofloxacin susceptibility in adult inpatients from 2010 to 2020.

**Fig. 3.**
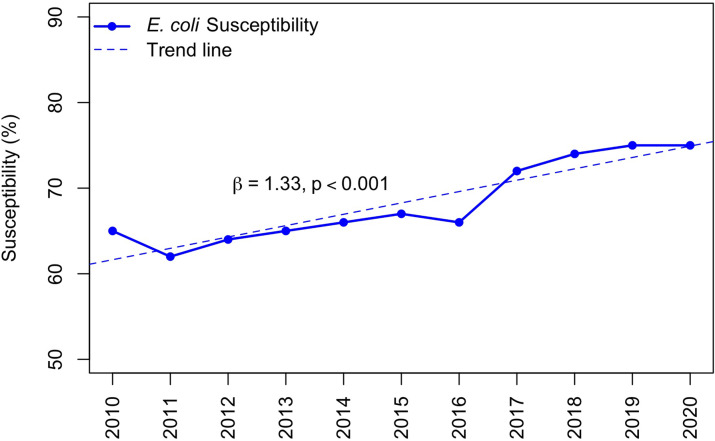
*E. coli* levofloxacin susceptibility in adult inpatients from 2010 to 2020.

Inpatient levofloxacin susceptibility increased for *P. aeruginosa* by 57% (absolute increase of 30%) and for *E. coli* by 15% (absolute increase of 10%) from 2010 to 2020. *P. aeruginosa* susceptibility to levofloxacin increased by an average of 2.73% per year, β = 2.73 (95% CI, 1.94–3.51; *P* < .001) (Fig. 2). We detected a strong negative association between fluoroquinolone use and *P. aeruginosa* susceptibility to levofloxacin, r = −0.99 (*P* = .002). *E. coli* susceptibility to levofloxacin increased by an average of 1.33% per year, β = 1.33 (95% CI, 0.91–1.75; *P* < .001) (Fig. 3). We also detected a strong negative association between fluoroquinolone use and *E. coli* susceptibility to levofloxacin, r = −0.95 (*P* = .015).

## Discussion

The ASP efforts at our health system appear to be successful at reducing inpatient fluoroquinolone use and antimicrobial resistance among *P. aeruginosa* and *E. coli* isolates.

The results of this study add to the growing body of evidence that demonstrates the importance of persistent and well-resourced hospital-based ASPs. Several meta-analyses have summarized favorable outcomes for individual stewardship interventions as well as full programs on antibiotic use and resistance, clinical outcomes, adverse events, *C. difficile* infections, and costs.^15–19^ For these reasons, the ASP at our health system is valued and has expanded to provide comprehensive services across the health system. The desirable trends in fluoroquinolone use and susceptibility reported here provide further justification for our program.

We detected a 74% decrease in fluoroquinolone use over 5 years and an absolute increase of 30% and 10% in levofloxacin-susceptible *P. aeruginosa* and *E. coli* isolates, respectively, over 10 years. ASP implementation is typically accompanied by an ∼20% decrease in total antimicrobial use, which is consistent with studies reporting on fluoroquinolone use specifically.^17,20,21^ Resistance reduction rates vary widely between studies.^22–25^ We detected a greater reduction in use and resistance than has typically been reported. Although it is difficult to compare results between studies with heterogeneous methods, one possible explanation may be the longer observation period in our study compared with shorter 1–2-year intervention periods in most reports. In fact, a greater magnitude of resistance reduction is observed when fluoroquinolone use and resistance is assessed over a longer period.^23,26^ Interestingly, we saw a numerically greater improvement in *P. aeruginosa* susceptibility than in *E. coli*. These results are consistent with other studies that describe either no impact on *E. coli* susceptibility in contrast to *P. aeruginosa* ^24,27^ or only marginal improvement.^22,25,28^

Our results provide support for non–restriction-focused ASP services. Most current evidence for ASP impact on gram-negative fluoroquinolone resistance includes formulary restriction as the main intervention.^13^ However, 2 studies have described interventions that included prospective audit and feedback without a restriction component.^24,29^ The interventions were successful in both studies, demonstrating a significant decrease in fluoroquinolone use and absolute increase of 9%–16% in fluoroquinolone-susceptible *P. aeruginosa* isolates over an ∼5-year period, which is in line with our results. Our study is unique because it not only provides fluoroquinolone susceptibility trends over 10 years but also has a longer stewardship intervention period than the 2 studies.

Another strength of our study is the utilization of order-set optimization and prospective audit and feedback as the core stewardship intervention, which can be adopted in virtually any hospital setting without the need for around-the-clock staff support, as might be required by some formulary restriction models. We might have observed even greater benefits if our system employed formulary restriction in addition to prospective audit and feedback and order-set optimization. When considering longitudinal antimicrobial stewardship activities, ASPs should periodically re-evaluate their approaches and adapt their activities based on needs and resources.

Antimicrobial stewardship activities at our health systems are primarily pharmacist driven, which is similar to other stewardship programs.^30^ Pharmacists, particularly those with infectious diseases expertise, are uniquely qualified to provide robust antimicrobial stewardship and therapeutic optimization.^31,32^ Leveraging pharmacists’ drug expertise appears to have been successful in reducing both antimicrobial use and antimicrobial resistance at our health system. In addition to empowering pharmacists to perform stewardship activities and promoting their expertise within our health system, investment in staffing likely contributes to the successes described. Various recommendations of ratios of antimicrobial stewardship staff to hospital beds exist, but our ratio of 1 full-time infectious diseases pharmacist to 400 beds may allow for more robust prospective audit and feedback and higher impact relative to ASPs with fewer staff members.^33^

This study had several limitations. Fluoroquinolone use data were not available for inpatients before 2016. How use correlated with susceptibility before this time remains uncertain, but other than the stewardship activities described, there were no other clinical interventions, operational changes, or changes in infection prevention practices that could have significantly influenced susceptibility. Based on first-hand knowledge of stewardship activities and prescribing patters prior to 2016, we know the use of fluoroquinolones was substantially higher in 2011 and decreased over time. We could not assess the impact of our program in a quasi-experimental study design due to lack of data prior to the intervention period. We also did not report use and antimicrobial resistance trends for other antibiotic classes, which may have inadvertently increased. However, this is unlikely given that our program did not focus solely on fluoroquinolone use but addressed other broad-spectrum antibiotics in parallel. Susceptibility interpretations for fluoroquinolones were designated based on CLSI breakpoints before the change in 2019.^34^ Application of updated breakpoints likely would have resulted in decreased susceptibility rates; however, this would have been a major confounding factor, and for the purposes of this review, utilizing a consistent breakpoint likely more accurately reflects trends over time.

In our health system, fluoroquinolone use substantially decreased and *P. aeruginosa* and *E. coli* levofloxacin susceptibility increased after the implementation of an ASP. These results demonstrate the value of stewardship services and highlight the effectiveness of an infectious diseases pharmacist–led ASP.
